# A New Postoperative Regimen after CXL and PRK Using Topical NSAID and Steroids on the Open Ocular Surface

**DOI:** 10.3390/jcm11144109

**Published:** 2022-07-15

**Authors:** Farhad Hafezi, Mark Hillen, Leonard Kollros, Jerry Tan, Shady T. Awwad

**Affiliations:** 1 Laboratory for Ocular Cell Biology, Center for Applied Biotechnology and Molecular Medicine, University of Zurich, 8006 Zurich, Switzerland; 2 ELZA Institute, 8953 Dietikon, Switzerland; mhillen@elza-institute.com (M.H.); lkollros@elza-institute.com (L.K.); 3 Faculty of Medicine, University of Geneva, 1205 Geneva, Switzerland; 4 Department of Ophthalmology, University of Southern California, Los Angeles, CA 90007, USA; 5 Department of Ophthalmology, Wenzhou Medical University, Wenzhou 325015, China; 6 Jerry Tan Eye Surgery, Singapore 228208, Singapore; jerrytan@jerrytan.com; 7 Department of Ophthalmology, American University of Beirut Medical Center, Beirut 1107, Lebanon; sawwad@hotmail.com

**Keywords:** photorefractive keratectomy, corneal cross-linking, slit lamp, CXL, keratoconus, laser surgery, refractive surgery, UV, ultraviolet light, non-steroidal anti-inflammatory drugs, NSAID, corneal surgery, ophthalmology, eye, vision

## Abstract

Corneal epithelium removal during photorefractive keratotomy (PRK), TransPRK, or corneal cross-linking (CXL) means that patients experience pain and inflammation after the procedure, which need to be carefully managed with topical drug regimens. One highly effective class of topical analgesics is non-steroidal anti-inflammatory drugs (NSAIDs), but these must be used carefully, as their use has been associated with delayed re-epithelialization and, in rare cases, corneal melting. However, our clinical experience has been that the concomitant use of topical corticosteroids obviates this risk. Here, we present a mechanistic explanation for our observations, our TransPRK and epithelium-off CXL protocols, and the postoperative medication regimens where topical NSAIDs are used in combination with topical steroid therapy during the first two postoperative days (where pain and inflammation levels are the highest). We detail the results of a single-center retrospective case analysis that examined eyes that underwent TransPRK (*n* = 301) or epithelium-off CXL (*n* = 576). Topical NSAID use in the first two postoperative days to control pain and inflammation after PRK/TransPRK or epithelium-off CXL, when used in combination with topical steroid therapy, does not appear to be associated with corneal melting or delayed epithelial healing. This approach may represent an improvement over current methods of handling post-surgical pain in procedures that require corneal epithelial debridement.

## 1. Introduction

Transepithelial photorefractive keratotomy (TransPRK) and epithelium-off corneal cross-linking (CXL) are commonly performed corneal procedures, where the first step of the surgery is to remove corneal epithelial cells, either mechanically or by using an excimer laser [1,2,3]. This preparatory step is followed by either excimer laser ablation to reshape the stroma (PRK/TransPRK), or by the saturation of the stroma with riboflavin, followed by irradiation with ultraviolet A light (CXL). Common consequences of epithelial removal are postoperative pain during the five- to seven-day period of re-epithelization and prolonged inflammation over weeks to months. A typical postoperative approach would be to place a bandage contact lens (which has been shown to increase the speed of re-epithelialization and reduce pain), topical lubricant therapy, and prophylactic topical antibiotic therapy (third- or fourth-generation quinolones) as the epithelium closes [4]. Once this is achieved, then topical steroid therapy (typically with fluorometholone or dexamethasone) is commenced to manage inflammation and postoperative haze [5].

Non-steroidal anti-inflammatory drugs (NSAIDs) are commonly used to treat inflammation and pain, and at first glance, would appear to be suitable to manage the peak levels of pain and inflammation that occur during the first 1–2 postoperative days. However, this approach is only taken with caution in clinical practice. Several reports have been published describing NSAID-induced corneal melting [6]—a rare but serious adverse event—and delays in corneal re-epithelialization [7], and this may be related to the fact that NSAID use has been associated with the upregulated expression of several matrix metalloproteinases [8]. Furthermore, topical NSAID therapy is analgesic, and this can lead to patients applying these drops multiple times per day as soon as they experience pain, rather than the twice-daily regimens prescribed. This frequent instillation may result in unreasonably high doses being applied to the eye. For these reasons, topical NSAID therapy has not formed part of most post-PRK, TransPRK, or CXL pain management regimens [9].

We developed a method that combines both NSAIDs and steroids in the first two days after these procedures, when pain and inflammation are at peak levels. Our empirical clinical experience has been that this gives patients the desired analgesic effect of NSAIDs, but without the drawbacks of delayed re-epithelization or even corneal melting. To validate this approach, we performed a single-center retrospective case analysis to determine the occurrence of corneal melting in eyes that underwent a procedure that involved an initial step of corneal epithelium removal (TransPRK or epithelium-off CXL), which received topical NSAID use in the first two postoperative days to control pain and inflammation after PRK/TransPRK or epithelium-off CXL.

## 2. Technique

For all procedures, preoperative topical anesthesia was administered in the waiting room, with one drop of oxybruprocaine hydrochloride (4 mg/mL, Théa Pharma SA, Schaffhausen, Switzerland), followed by one drop of tetracaine 1% (Théa Pharma SA, Schaffhausen, Switzerland), applied three times each over a 10-minute period. For the various epi-off CXL procedures, the application of anesthesia was adapted according to the protocol of choice.

### 2.1. TransPRK

All procedures were performed by a single surgeon (F.H.) using the SCHWIND Amaris 750S excimer laser platform (SCHWIND eye-tech-solutions GmbH, Kleinostheim, Germany). After the patient reclined on the excimer laser bed, the eye and periorbital region were thoroughly disinfected with sterile cotton wool buds soaked in octenidine hydrochloride (Octenisept, Schülke & Mayr GmBH, Norderstedt, Germany). After the placement of a lid speculum, a moistened Merocel sponge (Medtronic Inc., Minneapolis, MN, USA) was applied to the corneal surface, with slow, painting movements to avoid uneven surface humidification. Immediately afterward, laser ablation was performed, and the cornea was then cooled with a 20 mL chilled balanced salt solution. When the stromal ablation was more than 90 μm, mitomycin-C (0.02%; Medac GmbH, Wedel, Germany) was immediately applied for 30 s using a damp Merocel sponge, then copiously irrigated, and dried. Topical ketorolac (0.5%) and moxifloxacin (0.3%) were subsequently instilled, and a bandage contact lens (Acuvue Oasys; Johnson & Johnson, Jacksonville, FL, USA) was inserted.

### 2.2. Epithelium-Off CXL

The patient was brought to the slit lamp (SL9900, CSO Italia, Florence, Italy), where the height of the chair and slit lamp were adjusted to ensure the patient’s maximal comfort. This step is important, because once comfortably seated, the patient is able to keep a steady position more easily during irradiation. For that purpose, we also used a chair with two armrests, rather than a simple stool. While the patient was in the sitting position, the eye and periorbital region were thoroughly disinfected with sterile cotton wool buds soaked in octenidine hydrochloride. A lightweight open-wire speculum (Kratz speculum, enclosed in the C-Eye Procedure Kit, EMAGine AG, Zug, Switzerland) was inserted, and sterile surgical gauze was taped laterally to the temporal canthus to collect any riboflavin solution run-off.

### 2.3. Abrasion

For epi-off CXL, several different approaches can be used to remove the epithelium, such as by means of a hockey knife or an Amoils brush. However, these surgical tools may be challenging to maneuver in the upright position. Additionally, special attention should be taken not to injure the Bowman’s membrane during the epithelial removal process. Therefore, we used an alternative approach to remove the epithelium. This approach was a modified laser-assisted subepithelial keratectomy (LASEK) approach [10] using a sterile cotton swab soaked with 40% ethanol. The epi-off process using the cotton swab and 40% ethanol was easy to perform, without potential harm to the Bowman’s membrane, rapid, and safe to perform. Specifically, a sterile cotton swab was dipped in a freshly prepared 40% ethanol solution, then gently tapped on the center and periphery of the cornea in a circular fashion for 70 s. After approximately 45 s of tapping, a loosening and folding of the epithelium could be seen. After 70 s, gentle pressure was applied to the cornea with the cotton swab tip to wipe away the epithelium in a circular motion. An erosion of approximately 8 mm appeared. Particular care must be taken that the 40% ethanol is not exposed to the air for prolonged periods prior to use. The evaporation of the ethanol from the solution rapidly changes the ethanol content within the solution and, ultimately, the effectiveness of the epithelium removal process. Finally, the cornea was rinsed with a balanced salt solution (BSS) using a syringe with an irrigation cannula. For a PACK-CXL treatment, epithelial debris was removed over and/or around the infiltrate using a dry sterile triangular sponge.

### 2.4. Postoperative Medication

Our post-TransPRK/CXL medication scheme is depicted in Table 1. Briefly, we applied topical Tobradex (0.1% tobramycin–0.3% dexamethasone, Novartis Pharma, Basel, Switzerland) and Vigamox (moxifloxacin 0.5%; Alcon, Geneva, Switzerland) immediately in the postoperative period and for the first post-surgical week. This means that the patient received topical steroid therapy from the start, and topical NSAID therapy (Acular, Ketorolac trometamol 5 mg/mL, Allergan Inc., Zurich, Switzerland) was administered twice-daily for the first two days, before its use was discontinued. We then continued with Vigamox and Tobradex on the open surface for the next 4 days (until the corneal epithelium had closed). After this, fluorometholone 0.1% eye drops (FML Liquifilm, Allergan) were administered twice a day for up to 4 weeks after surgery. We also prescribed preservative-free, single-dose artificial tears ad libitum (Povidon K25 50 mg/mL, unit dose, preservative-free [Allergan]), oral vitamin C as a free radical scavenger for the first postoperative week, and ibuprofen and paracetamol for the first two postoperative days.

### 2.5. Pain Questionnaire

A subset of patients was asked to complete a pain questionnaire within the first two postoperative days, in which the patients were asked to rate their ocular pain from a scale of 0 (no pain at all) to 10 (worst pain imaginable), according to the visual analogue scale [11].

At 4 weeks after surgery, corneal haze was determined both clinically at the slit lamp and by using corneal densitometry. Depending on the grade of haze, we either continued with fluorometholone 0.1% eyedrops or switched to loteprednol etabonate 0.5% or preservative-free dexamethasone 0.1% eye drops for another 8 weeks.

## 3. Results

In the period between 11 January 2016 and 29 April 2022, a single surgeon (F.H.) at the ELZA Institute, Zurich, performed TransPRK on 301 eyes and epithelium-off CXL on 576 eyes (total: 877 eyes), all of which received the postoperative medication regimen detailed in Table 1.

Epithelial closure usually occurred within 4 to 5 days. Not a single patient experienced a corneal melt, nor were any instances of delayed corneal re-epithelialization (≥8 days) noted. Two cases of a suspected beginning sterile infiltrate were noted, but all resolved within 10 days, and no cases of infectious keratitis were observed. Pain questionnaire scores were available for 77 eyes, with the mean pain score (standard deviation) being 0.85 (1.77).

## 4. Discussion

**Figure 1:**
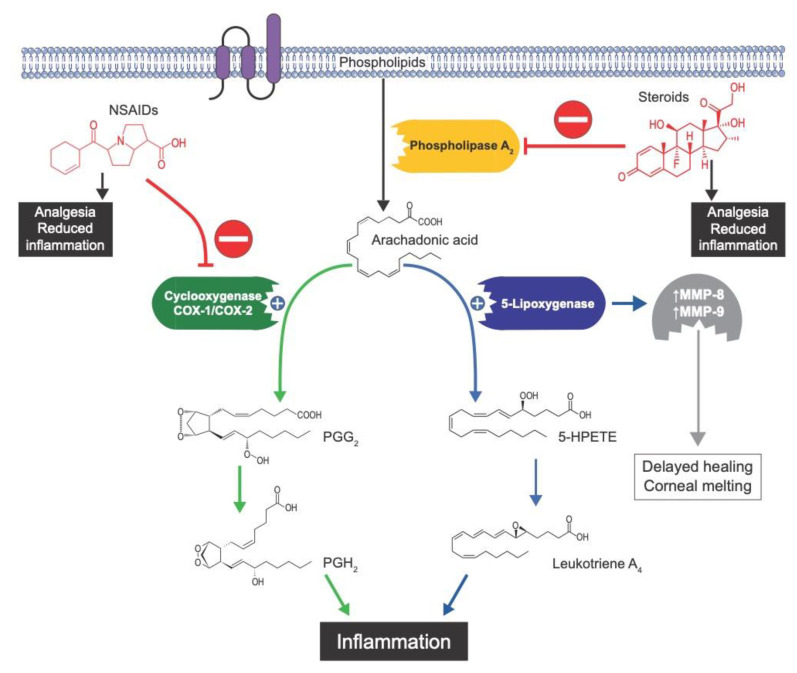
NSAIDs deliver effective analgesia through inhibition of the cyclooxygenase enzyme family, which pushes inflammation down the 5-lipoxygenase pathway, which ultimately results in upregulation of matrix metalloproteinases (MMPs) that can digest the stroma and cause melt formation. Steroids inhibit phospholipase A_2_, the enzyme above both COX and 5-lipoxygenase in the inflammation cascade. From a mechanistic perspective, steroid use may prevent melting that occasionally occurs with topical NSAID use. 5-HPETE: arachidonic acid 5-hydroperoxide; MMP: matrix metalloproteinase; NSAID: non-steroidal anti-inflammatory drug; PG: prostaglandin.

Inflammation can be caused by two main pathways: the cyclooxygenase (COX) pathway and the lipoxygenase pathway (Figure 1) [12]. Most NSAIDs block COX activity, meaning that the inflammation present is therefore driven by lipoxygenase, which can upregulate matrix metalloproteinase-9 (MMP-9) activity. Elevated MMP-9 levels may, in return, lead to corneal melting. Ideally, the surgeon would simultaneously administer NSAIDs and a topical lipoxygenase inhibitor to ensure that both arms of the inflammatory cascade are blocked. However, topical lipoxygenase inhibitors are not available to date. Steroids, however, inhibit phospholipase A_2_ [13], located upstream of lipoxygenase in the inflammatory pathway (Figure 1), and there are several topical ophthalmic steroid drugs to choose from. From a mechanistic perspective, the concomitant use of steroids with NSAIDs therefore blocks both COX and lipoxygenase, inhibiting both arms of the inflammatory pathway, which should stop melting from occurring and help explain the favorable results seen in our study.

A twice-daily administration of topical NSAID therapy during the first two postoperative days after PRK, TransPRK, or epithelium-off CXL surgery provides patients with effective pain and inflammation prophylaxis. This, combined with topical steroid therapy, appears to obviate the risk of corneal melt formation and delayed re-epithelialization that has been associated with the increased lipoxygenase and matrix metalloproteinase activity that can occur when NSAIDs are used in the absence of steroid therapy. This approach may not be suitable for all patients, such as those with autoimmune diseases, such as rheumatoid arthritis or Sjögren’s syndrome, who may already be receiving high-dose oral NSAID therapy. In these patients, the conventional post-surgical drug regimen may be more appropriate, or in the case of eyes requiring cross-linking, epithelium-on CXL protocols should be considered.

This study has limitations. The study was a large, retrospective case series, and because the protocol described above was routine clinical practice at the study site, there was no “traditional” pain relief population to act as a control group, nor were pain assessment questionnaires issued routinely to patients. While we did not experience any NSAID-related adverse events, and the risks related to this combined approach appear to be minimal, a formal clinical trial comparing patient outcomes using standard-of-care and this novel approach, including comprehensive pain assessments, would be worthwhile, as would continued outcome surveillance.

## 5. Conclusions

Topical NSAID use in the first two postoperative days to control pain and inflammation after PRK/TransPRK or epithelium-off CXL, when used in combination with topical steroid therapy, appears not to be associated with corneal melting or delayed epithelial healing. Given that this approach provides patients with effective pain relief during the period of peak postoperative pain, this regimen could represent an improvement over current methods of handling post-surgical pain in procedures that require corneal epithelial debridement, such as PRK and CXL.

## Figures and Tables

**Table 1:** Post-PRK/TransPRK/CXL medication regimens employed during the study.

	Day of the Operation/Days 1 and 2 Afterwards													Days 3–6 after Surgery													Day 7 until End of Week 12
Time	0800	0900	1000	1100	1200	1300	1400	1500	1600	1700	1800	1900	2000	0800	0900	1000	1100	1200	1300	1400	1500	1600	1700	1800	1900	2000	
**Topical (mandatory)**																											
Antibiotic (moxifloxacin 0.5% solution)	×				×				×				×	×				×				×				×	Stop
Antibiotic with steroid (tobramycin 3 mg/mL and dexamethasone 1 mg/mL ophthalmic suspension)	×				×				×				×	×				×				×				×	Stop
NSAID (ketorolac trometamol 5 mg/mL ophthalmic solution)			×								×			Stop													
Steroid (fluorometholonum 1 mg/mL solution)														Start on day 7													
														×										×			Stop
Antibiotic (Ofloxacin 0.3% eye ointment)														Start only when bandage contact lens is removed (day 3 or 4)													
														×		×		×		×		×		×			Stop
Artificial tears (Povidon K25 50 mg/mL)	×	×	×	×	×	×	×	×	×	×	×	×	×	×	×	×	×	×	×	×	×	×	×	×	×	×	Stop
**Oral (mandatory)**																											
Vitamin C 1000 mg dispersible tablet	×													×													Stop
Ibuprofen 400 mg tablet	×						×						×	Stop													
Paracetamol 500 mg tablet	×						×						×	Stop													
**Oral (optional)**																											
Tramadol 50 mg capsule	×				×				×				×	Stop													
Anti-emetic: Meclozine dihydrochloride [25 mg], Caffeine [25 mg], and Pyridoxine hydrochloride [25 mg] capsule	×				×				×				×	Stop													

CXL, corneal cross-linking; NSAID, non-steroidal anti-inflammatory drug; TransPRK, transepithelial photorefractive keratotomy.

## Data Availability

The data underlying this article will be shared at reasonable request to the corresponding authors.
